# Massive gastrointestinal haemorrhage from a duodenal diverticulum: a case report

**DOI:** 10.4076/1757-1626-2-6710

**Published:** 2009-07-02

**Authors:** Sanjeewa Anuruddha Seneviratne, Dharmabandu Nandadeva Samarasekera

**Affiliations:** University Professorial Surgical Unit, National Hospital of Sri LankaColombo 08Sri Lanka

## Abstract

Bleeding duodenal diverticulum is a rare cause of gastrointestinal bleeding, which sometimes can be massive and life threatening. We report one such case and the management, with a successful outcome. Different diagnostic techniques and management options available are also discussed.

## Introduction

Duodenum is the commonest site of diverticulae in the small bowel with a reported prevalence rate of up to 25% in autopsy studies [1]. The majority of them are asymptomatic and usually identified as an incidental finding at endoscopy or in barium studies. Complications from duodenal diverticulae occur due to mechanical pressure effects or due to diverticulitis [2].

## Case presentation

A 54 year old previously healthy Sri Lankan, ethnic Sinhalese male was admitted with a two day history of haematemesis and melaena. There were no other gastrointestinal symptoms. On examination the patient was extremely pale with tachycardia and hypotension. The abdominal examination was unremarkable.

After initial resuscitation with multiple blood transfusions, an upper GI endoscopy was performed. It showed bleeding from the medial wall of the second part of the duodenum, from a duodenal diverticulum with a narrow neck. Attempts of endoscopic haemostasis failed since the endoscope couldn’t be negotiated through the narrow neck in to the diverticulum, to identify and arrest the bleeder deep inside the diverticulum.

**Figure 1.**
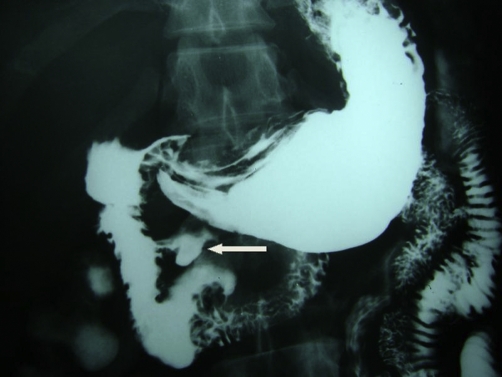
Barium meal and follow through demonstrating the diverticulum (indicated by white arrow) arising from the second part of the duodenum.

**Figure 2.**
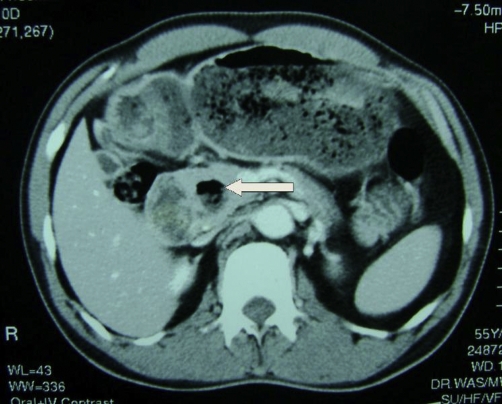
Contrast enhanced CT scan demonstrating the diverticulum (indicated by white arrow) extending towards the head of the pancreas.

Barium meal and follow through (Figure 1- Barium meal and follow through demonstrating the diverticulum (arrow) arising from the second part of the duodenum) and contrast enhanced CT scan (Figure 2- Contrast enhanced CT scan demonstrating the diverticulum (arrow) extending towards the head of the pancreas) of the abdomen confirmed the presence of a duodenal diverticulum arising from the medial wall of the second part of the duodenum.

**Figure 3.**
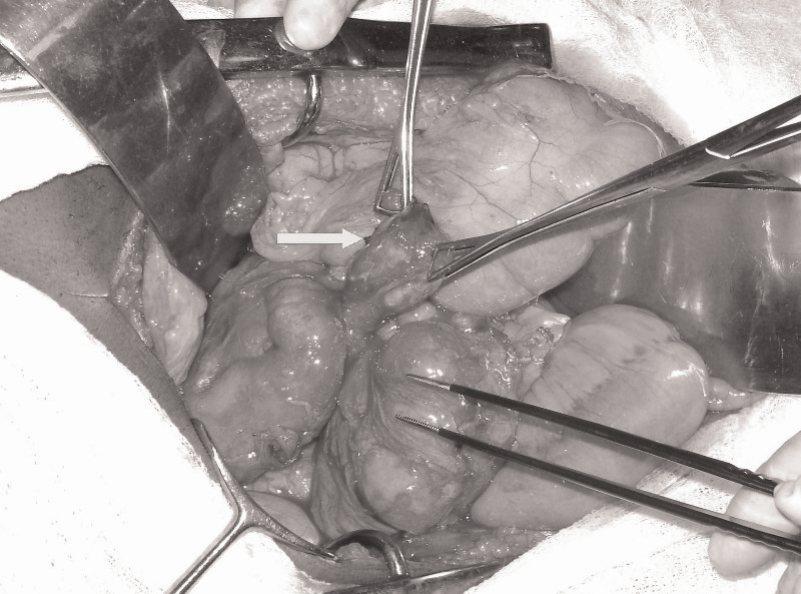
Diverticulum (indicated by white arrow) arising from the medial wall of the duodenum.

At surgery, a diverticulum was seen arising from the postero-medial wall of the duodenum extending to the left, behind the head of the pancreas (Figure 3- Diverticulum (arrow) arising from the medial wall of the duodenum). Two large veins (probably the source of bleeding), were seen on the wall of the diverticulum. Diverticulectomy and primary repair with non absorbable sutures was carried out.

Histology showed a false diverticulum containing only the mucosa and submucosa in its wall.

The patient had no postoperative complications and was discharged 5 days later. He remained free of symptoms with a normal endoscopy six months later.

## Discussion

Duodenal diverticulae are acquired, and consist of a sac of mucosal or sub mucosal layers herniating through a muscular defect in bowel wall, but the precise manner of development is not known. Over 95% of duodenal diverticulae project from inner or pancreatic border of duodenal curve in second, third and fourth parts with the second part being the commonest site [3].

Only about 10% of duodenal diverticulae produce symptoms, such as epigastric pain, nausea and vomiting. Pressure effects may give rise to jaundice, cholangitis, pancreatitis and obstruction of the duodenum or pancreatic or bile ducts [4,5]. Inflammation giving rise to diverticulitis can lead to perforation, ulceration, haemorrhage and abscess formation.

The reported incidence of bleeding from a duodenal diverticulum is about 7% [6]. The majority of diverticulae can be diagnosed by upper GI barium studies and endoscopy, especially by side viewing endoscopy. It may be necessary to perform both investigations since the failed visualization of the diverticulum with one investigation does not exclude the presence of a duodenal diverticulum [7].

Treatment options include endoscopic haemostasis, endoscopic incision and ligation of the diverticulum, embolization and surgery [8-10]. Usefulness of endoscopic techniques as a definitive procedure is limited due to the high incidence of re-bleeding [11]. Successful super-selective arterial embolization has been described, but it is technically demanding [12]. Surgery remains the definitive treatment in such cases. Duodenal diverticulectomy is an effective procedure but it is associated with a considerable leak rate of 30 to 50% [4,6]. It can be undertaken either as an open or laparoscopic procedure.

In our patient open surgery was undertaken with preparation for a possible pancreatico-duodenectomy, but a simple diverticulectomy was possible after separating the diverticulum from the pancreatic head.

## Abbreviations

CT: Computer Tomography
GI: Gastrointestinal
